# Optimizing a Conventional Multiplex PCR for Simultaneous Detection of Granulomatous Skin Infection Agents: *Leishmania aethiopica*, *Mycobacterium leprae*, and *Mycobacterium tuberculosis*

**DOI:** 10.1155/jotm/1456781

**Published:** 2026-03-11

**Authors:** Selfu Girma, Mesfin Gemeda, Adugna Woldesemayat, Dawit Alemayehu, Dinksira Deneke, Semira Mekonen, Shimelis Doni, Hanna Beliye, Feleke Tilahun Zewdu, Tsegaye Kumssa, Tizita Kidane, Menberework Chanyalew, Almeseged Abdissa, Markos Alemayehu, Kidist Bobosha, Endalamaw Gadisa

**Affiliations:** ^1^ Armauer Hansen Research Institute, Addis Ababa, Ethiopia, ahri.gov.et; ^2^ Biotechnology and Bioprocess Centre of Excellence, Addis Ababa Science and Technology University, Addis Ababa, Ethiopia, aastu.edu.et; ^3^ Department of Pathology, School of Medicine, Addis Ababa University College of Health Science, Addis Ababa, Ethiopia; ^4^ Dermatology Unit, ALERT Comprehensive Specialized Hospital, Addis Ababa, Ethiopia; ^5^ Boru Meda General Hospital, Dessie, Amhara Region, Ethiopia; ^6^ Department of Dermatology & Venerology, School of Medicine, School of Medicine, Addis Ababa University College of Health Science, Addis Ababa, Ethiopia

**Keywords:** cutaneous leishmaniasis, granulomatous skin infections, leprosy, molecular diagnosis, skin tuberculosis

## Abstract

**Background:**

Conventional polymerase chain reaction (PCR) assays are well‐established molecular techniques that can be integrated as standard diagnostic tools, especially in referral settings. This study aimed to assess the diagnostic potential of a multiplex PCR (mPCR) assay for the diagnosis of cutaneous leishmaniasis (CL), skin tuberculosis, and leprosy.

**Method:**

A cross‐sectional study was carried out involving 62 patients in the study group, comprising 45 with CL, 9 with leprosy, 4 with skin tuberculosis, and 4 with coinfections. Additionally, 112 positive control DNA samples were analyzed, including 37 of *M. tuberculosis*, 46 of *M. leprae*, and 29 of *L. aethiopica*. The study assessed sensitivity, specificity, positive predictive value (PPV), negative predictive value (NPV), and detection limits.

**Results:**

Sensitivity and specificity of the mPCR on positive and negative control samples were 100% (95% CI: 96.8%–100%) and 100% (95% CI: 94.9%–100%), respectively. Its sensitivity and specificity among the study group were 75.8% (95% CI: 63.3%–85.8%) and 100% (95% CI: 94.9%–100.0%), respectively.

**Conclusions:**

With further validation on more clinical suspects, mPCR has the potential to facilitate diagnosis in settings with coendemic CL, leprosy, and skin tuberculosis.

## 1. Introduction

Delays in diagnosis and treatment of skin disorders can exacerbate disability, lead to life‐threatening conditions such as sepsis, and contribute to social and mental health issues stemming from esthetic damage [1, 2]. Granulomas are commonly encountered inflammatory reaction patterns, exhibiting significant variability in their morphological characteristics, causes, clinical implications, and prognostic importance [3]. Granulomatous skin infections are caused by a variety of microorganisms, including bacteria, fungi, and parasites, triggering the formation of granulomas [4]. These conditions present challenges for dermatologists and pathologists in achieving a conclusive diagnosis essential for management decisions, due to their similar clinical features and histopathological patterns [5–10].

In Ethiopia, granulomatous skin infections hold a high portion of dermatology reports among all age groups [5, 11]. Cutaneous leishmaniasis (CL) is one of the major skin diseases, with an estimated annual incidence of 50,000 new cases, and over 29 million people living at risk [12, 13]. With over 2500 new cases annually, leprosy remains a public health importance [14], and skin tuberculosis is another frequently reported infection [15]. In a preliminary study in Ethiopia, of the 2342 histopathology reports, skin infections accounted for 560 (24%) with the three most common being 141 CL (25%), 138 skin tuberculosis (24.6%), and 112 leprosy (20%) [5].

The diagnosis of skin conditions in Ethiopia is primarily based on clinical presentations, which becomes even more challenging due to the limited number of dermatologists available [16]. Definitive diagnosis is made mainly through microscopic identification of the etiologic agents, despite the varied sensitivity [17–20]. In leprosy diagnosis, it varies from 31% to 78% depending on the stage and type of the disease [21], and in CL, it is usually between 50% and 70% [22]. Some research indicates that microscopic examination for acid‐fast bacilli (AFB) in skin tuberculosis lesions consistently yields negative results, despite supporting evidence from culture and/or histopathological analysis [23]. Culturing is another alternative method for diagnosing CL and skin tuberculosis; however, it is not easy to implement in resource‐limited settings and has a lengthy turnaround time, particularly for skin tuberculosis [24].

Molecular diagnostics demonstrate enhanced sensitivity compared to microscopy and culture methods when it comes to diagnosing skin diseases [25, 26]. Conventional polymerase chain reaction (PCR) is a technique that can easily be adapted as a point‐of‐care tool, especially in referral settings [27–29]. Different genomic targets are recommended for their potential application for leprosy, CL, and skin tuberculosis in a singleplex assay [30–33]. Repetitive elements of *Mycobacterium leprae* (RLEP), internal transcribed spacer‐1 (ITS‐1) of *Leishmania*, and the internal fragment of the mtp40 gene of *Mycobacterium tuberculosis* are among the widely evaluated targets with commendable sensitivity and specificity [34–38].

This study developed a conventional multiplex PCR (mPCR) assay and evaluated the diagnostic performance and detection limit in the possibility of coinfections.

## 2. Material and Methods

### 2.1. Study Design

**FIGURE 1:**
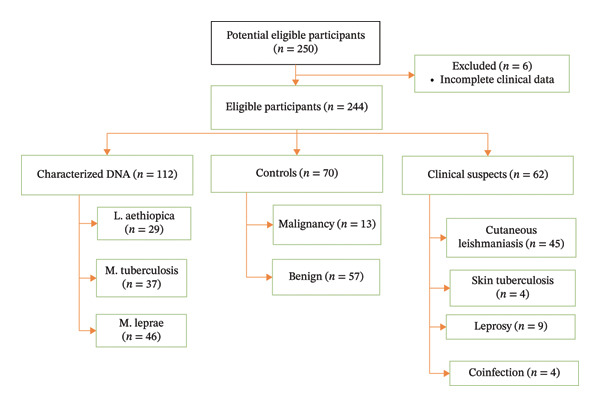
Flowchart of the study participants.

A cross‐sectional study was conducted from March 2023 to February 2024 at two dermatology referral hospitals. Convenience sampling was used to collect skin biopsy samples from 70 negative controls and 62 study group patients with CL (*n* = 45), leprosy (*n* = 9), skin tuberculosis (*n* = 4), and coinfections (*n* = 4) (see Figure 1). Additionally, stored positive control DNA samples were utilized, comprising 37 of *M. tuberculosis*, 46 of *M. leprae*, characterized stored DNA extracted from clinical samples in a previous stud*y,* and 29 of *Leishmania aethiopica* [34, 39, 40].

### 2.2. PCR Conditions and Gel Electrophoresis

In silico assessments were performed to evaluate the PCR conditions for potential crosslinking that might interfere with multiplexing, along with laboratory optimization techniques based on previous studies [31, 34].

The optimized cycling conditions included an initial denaturation at 95°C for 5 min, followed by 40 cycles consisting of denaturation at 95°C for 30 s, annealing at 54°C for 45 s, and elongation at 72°C for 40 s, with a final elongation step at 72°C for 10 min. The final reaction volume was set at 25 μL, which included 0.5 μL of 10 pmol of each forward and reverse primer for *M. leprae* and *M. tuberculosis*, along with 1 μL of 10 pmol of each primer for *Leishmania* (Supporting Table 1-A). The reaction incorporated 12.5 μL of HotStartTaq Master Mix (2X stock), following the supplier’s instructions. For all experimental reactions, 3 μL of the DNA template was added, and the final volume was adjusted with molecular‐grade water to reach the total of 25 μL.

The PCR product was visualized on 1.5% agarose gel stained with SYBR Safe. The electrophoresis was run for 50 min adjusted at 100V and 80 mA using a gel electrophoresis apparatus. The three visible DNA bands were identified as 450 bp, 328 bp, and 223 bp corresponding to M. *leprae*, *L. aethiopica*, and *M. tuberculosis*, respectively.

### 2.3. Clinical Classification of Leprosy

The clinical classification of leprosy was performed following WHO guidelines and categorized cases into two main groups based on clinical features for treatment purposes: paucibacillary (PB) and multibacillary (MB). PB leprosy is characterized by having up to five skin lesions, while MB leprosy involves more than five skin lesions [41].

### 2.4. Nontuberculous Mycobacteria (NTM) and Other *Leishmania* Strains

Other leishmania and NTM strains, *L. donovani* (MHOM/IN/80/DD8), *L. tropica* (MHOM/SU/74/K27), *L. infantum* (MHOM/TN/80/IPT‐1), *M. smegmatis* (NCTC10265, TMC1546). *M. terrae* (NCTC10856, TMC1450), *M. avium* (SSC1336, TMC724), and *M. fortuitum* (TMC1529), were used to check specificity.

### 2.5. Positive Control Samples

DNA samples of *L. aethiopica*, *M. tuberculosis*, and *M. leprae* from patients with CL, leprosy, and TB lymphadenitis were used [34, 39, 40]. To determine detection limits for single infections and potential coinfections, known DNA concentrations from reference isolates of *L. aethiopica* (MHOM/ET/72/L100) and *M. tuberculosis* (TMC102 H37Rv), along with a clinical sample of *M. leprae* (high bacterial index: 6+), were pooled (see Supporting Table 1-B).

### 2.6. Negative Control Skin Sample

A skin sample measuring approximately 10 mm by 10 mm was obtained from patients without leprosy, skin tuberculosis, or leishmaniasis for molecular testing.

### 2.7. Skin Sample From the Study Group

Dermatologists evaluated participants clinically to determine and include participants in the study. A 3‐mm skin punch biopsy was collected with standard procedure and used for molecular analysis [42].

### 2.8. DNA Extraction and Quality Control

DNA extraction was performed using the DNeasy Blood & Tissue Kit according to the supplier’s protocol, with the modification that DNA was eluted in 100 μL of elution buffer instead of the standard 200 μL. [34, 43]. To confirm successful DNA extraction, a PCR was conducted to amplify a 304‐bp product of the human β‐actin gene using β‐actinF and β‐actinR primers (Supporting Table 1-A) [44].

### 2.9. Statistical Analysis

Stata Version 17 was utilized to assess sensitivity, specificity, negative predictive value (NPV), and positive predictive value (PPV), along with 95% confidence intervals. The clinical diagnosis was considered the gold standard for both the negative control and study groups, while a combination of previous positive results from AFB staining, Giemsa staining, H&E staining, or culture results was used as the gold standard for the positive control.

## 3. Results

### 3.1. Age and Sex Distribution

The average age of the 244 participants was 31.7 years ( ± 14.8), with 60.7% (*n* = 148) identifying as male and 39.3% (*n* = 96) as female. Among the groups, the positive control had 112 participants, the negative control consisted of 70 participants, and the study group included 62 participants. The male proportions were 56.3% (*n* = 63) in the positive control, 51.4% (*n* = 36) in the negative control, and 79.0% (*n* = 49) in the study group. The average ages for the study, negative control, and positive control groups were 29.2 years ( ± 13.9), 33.4 years ( ± 17.6), and 33.2 years ( ± 14.5), respectively.

### 3.2. Clinical Characteristics of Negative Controls Participants

Among the 70 participants in the negative control group, skin disorders were divided into two primary categories: malignant, accounting for 18.6% (*n* = 13), and benign, which made up 81.4% (*n* = 57). For malignant conditions, melanoma was the most common, representing 8.6% (*n* = 6). The benign disorders mainly consisted of chronic wounds not associated with the three granulomatous skin infections in this study, comprising 14.3% (*n* = 10) (Supporting Table‐2).

### 3.3. Site of Skin Lesions of the Study Group

Leprosy and skin tuberculosis affected various anatomical sites, including the breast, gluteal region, face, and abdomen. In contrast, CL primarily affected the forearm, with 10 cases (22.2%). The nose and face followed, each with 6 cases (13.3%). The forehead and ear had 5 cases (11.1%) each, while the leg, neck, and lip accounted for 3 (6.7%), 2 (4.4%), and 1 case (2.2%), respectively. Additionally, 7 cases (15.6%) of CL involved multiple sites, including the face, forearm, trunk, leg, and forehead (Supporting Table 4).

### 3.4. NTM and Other *Leishmania* Strains

The mPCR was evaluated for the detection of other *Leishmania* and NTM reference strains available in our laboratory. While the genus‐specific primer was positive for all four reference strains of *Leishmania*, the primer amplifying the mtp40 gene gave a positive result only for *M. tuberculosis* (Supporting Figure 2A).

### 3.5. Sensitivity, Specificity, PPV, and NPV in the Control Group

The sensitivity and PPV of the mPCR were both found to be 100% (95% CI: 96.8%–100%) across all 112 positive control samples and 70 negative control samples. Similarly, the specificity and NPV were also found to be 100% (95% CI: 94.9%–100%) (Supporting Figure 2B).

### 3.6. Detection Limits of the Assay

The detection limit in the simulated coinfection was established by examining three visible bands, revealing 0.3 ng of *L. aethiopica* and 0.01 ng of *M. tuberculosis* at the final dilution (Supporting Figure 2C). For double infections, the limits were also 0.3 ng for *L. aethiopica* and 0.01 ng for *M. tuberculosis* (Supporting Figure 1-A). In single infection of *L. aethiopica*, the detection limit remained similar, with no significant change in band visibility. Further dilution of *M. tuberculosis* showed a final visible band at 5 pg DNA per reaction (Supporting Figure 1-B).

### 3.7. Sensitivity, Specificity, PPV, and NPV in the Study Group

Diagnostic performance of mPCR on 62 study group samples prevailed sensitivity and specificity of 75.8% (95% CI: 63.3%–85.8%) and 100% (95% CI: 94.9%–100.0%), respectively. Furthermore, the PPV and NPV were 100% (95% CI: 92.5%–100.0%) and 82.4% (95% CI: 75.0%–87.9%), respectively (Supporting Figure 2D and Supporitng Table 3).

Given that CL was the most common condition in the study group, the performance mPCR was assessed separately for 45 clinical cases of CL. The results showed a sensitivity of 75.6% (95% CI: 60.5%–87.1%), a specificity of 100% (95% CI: 94.9%–100.0%), a PPV of 100% (95% CI: 90.0%–100.0%), and a NPV of 86.4% (95% CI: 79.2%–91.4%). Additionally, mPCR detected positivity in 100% (*n* = 9) of leprosy cases and 50% (*n* = 2) of cutaneous tuberculosis cases.

In the case of two samples suspected of having coinfections of skin tuberculosis and CL, the mPCR test yielded a positive result for skin tuberculosis in one sample, while the other sample returned a negative result. Additionally, among the two cases diagnosed with CL and leprosy coinfections, one tested positive for CL using mPCR, while the other was negative.

## 4. Discussion

**FIGURE 2:**
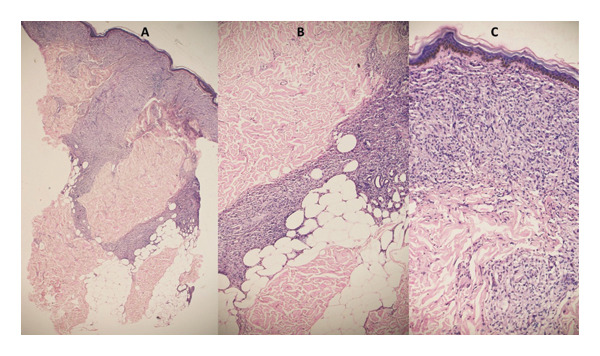
H&E staining of a histological section from a skin biopsy of an mPCR‐confirmed leprosy (positive control) participant. A (40X), B (100X), and C (200X) magnifications demonstrate diffuse infiltration of mostly foamy histiocytes and lymphocytes occupying the papillary and reticular dermis.

**FIGURE 3:**
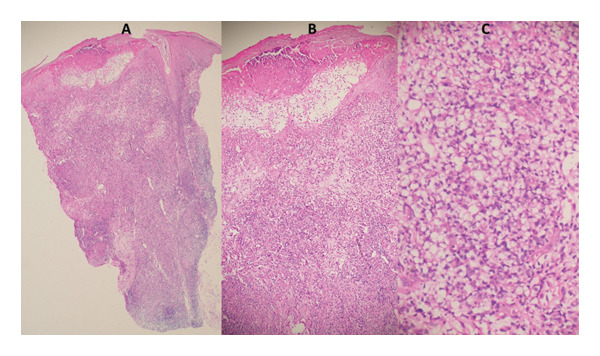
H&E staining of a histological section from a skin biopsy of a PCR‐confirmed cutaneous leishmania (positive control) participant. A (40X), B (100X), and C (400X) magnifications display ulceration of the epidermis and dermis represented having lymphocytic and plasmacytic inflammation accompanying with epithelioid granuloma and intracellular LD bodies.

In Ethiopia, granulomatous skin infections are mainly caused by *M. leprae*, *M. tuberculosis*, and *L. aethiopica* [5]. Healthcare settings remote from referral facilities primarily rely on clinical diagnosis [8]. Nevertheless, even experienced dermatologists struggle to report a conclusive diagnosis in some cases due to the overlapping clinical and histopathological features of these diseases [6, 45, 46]. The routinely used laboratory diagnostic methods, microscopy and culture, are either less sensitive or unavailable in all circumstances [24, 47]. Histopathology has considerable power for differential diagnosis when combined with clinical characteristics (see Figures 2 and 3) [48]. However, its limited sensitivity in detecting scanty etiologic agents is a common drawback [17, 49].

Molecular tests are usually sensitive for the diagnosis of different types of skin infections and are reported to be more appropriate for granulomatous skin infections [23, 50]. Conventional PCR is an ideal diagnostic tool for resource‐limited settings because it requires relatively less specialized expertise and advanced technology [28, 51]. In this work, the mPCR of three DNA targets, RLEP, ITS‐1, and mtp40, was assessed for the simultaneous diagnosis of leprosy, CL, and skin TB. These DNA targets have been intensively evaluated in different countries, including Ethiopia, and reported to be sensitive and specific in the singleplex assay [31, 34, 38, 52].

**FIGURE 4:**
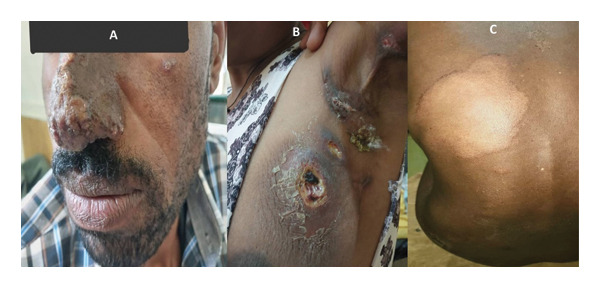
The clinical presentations of study participants: (A). MCL: indurated plaque with crustation over the nose extending to the nasal mucosa, (B) skin tuberculosis: erythematous indurated plaque with ulceration and purulent discharge, (C) Leprosy: hypopigmented patch over the trunk with peripheral erythematous induration.

In this current study, a total of 112 positive control DNA samples, 70 negative control skin samples, and 62 skin biopsy samples from the study group comprising CL, leprosy, and skin tuberculosis were utilized (see Figure 4). Our result prevailed that the mPCR had a sensitivity and specificity of 75.8% (95% CI: 63.3%–85.8%) and 100% (95% CI: 94.9%–100.0%), respectively. Furthermore, its detection limits for *M. tuberculosis* and *L. aethiopica* were found to be 5 pg and 0.3 ng, respectively. Additionally, *Leishmania* strains with unique RFLP characteristics were identified that require additional molecular studies to see to the association with severity of the disease and treatment outcome.

Even though nine out of ten suspected leprosy and coinfection case patients were reported to be positive, the assay only detected three of the six clinically suspected skin tuberculosis and potential coinfection cases. Furthermore, due to the absence of *M. leprae* reference standard DNA in our laboratory, and as the DNA sample extracted from clinical samples contains the host DNA, we were unable to determine the analytical sensitivity of the assay for *M. leprae*.

Focusing on the same RLEP region, our previous research evaluated a singleplex conventional PCR for leprosy diagnosis using a substantial number of clinical samples, achieving a sensitivity of 91.1% [34]. Although the small number of leprosy patients in the current study is a limitation, it demonstrates that the mPCR operates effectively. In addition to the 89.4% sensitivity of a previous study of ITS‐1 PCR on clinical samples of *Leishmania*, the detection limit was 0.1 ng [31]. Comparatively higher sensitivity was reported in this earlier work, which is primarily related to the utilized splenic aspirate sample, which has been shown to provide 93%–99% sensitivity in other studies [53]. The current study found a sensitivity of 75.6% and a similar detection limit of 0.3 ng for cutaneous leishmania. A study in India reported that the mtp40 gene detection of *M. tuberculosis* in extra pulmonary tuberculosis cases was more sensitive than the IS6110 region, despite the lower sensitivity of 41.6% [52]. The primers might exhibit reduced sensitivity since they are only capable of detecting the target within the *M. tuberculosis* complex.

A limitation of this study was the small sample size for leprosy, skin tuberculosis, and potential coinfections. We recommend that future research include larger, well‐powered samples from these groups before clinical adoption. Developing probe‐based multiplex qPCR assays will also help identify other causative agents of granulomatous skin infections and enhance sensitivity in PB cases of leprosy and tuberculosis. Additionally, since relying only on clinical diagnosis as the gold standard restricts the definitive validation of the mPCR results, adding microbiological or other available immunologic testing would help strengthen the overall the findings. For determining the analytical sensitivity for *M. leprae*, we also recommend the future studies to use international reference DNA of *M leprae*.

## 5. Conclusions

By validating additional clinical samples from diverse regions, mPCR could become a routine diagnostic tool for CL, skin tuberculosis, and leprosy. Clinicians might also use mPCR as an alternative differential diagnostic test, especially where these infections co‐occur. This could enhance diagnostic accuracy and improve patient management in affected communities.

## Funding

No funding was received for this manuscript.

## Conflicts of Interest

The authors declare no conflicts of interest.

## Supporting Information

Additional supporting information can be found online in the Supporting Information section.

## Supporting information


**Supporting Information 1** Supporting Table 1. List of primers utilized in this study (1A) and serial dilution of the analysis of the detection limits of the assay (1B).

**Supporting Information 2** Supporting Table 2. Diagnosis of the negative control group.

**Supporting Information 3** Supporting Table 3. Cross‐tabulation of the study and negative control groups.

**Supporting Information 4** Supporting Table 4. Clinical data of study participants.

**Supporting Information 5** Supporting Figure 1. Detection limits of the assay in potential double infections (1‐A), single infections (1‐B), and ITS‐1 product following digestion with the Hae‐III restriction enzyme (1‐C).

**Supporting Information 6** Supporting Figure 2. Agarose gel electrophoresis (1.5%); A. Specificity of the assay on Mycobacterial and Leishmania reference strains. B. Characterized DNA samples of *M. tuberculosis*, *L. aethiopica*, and *M. leprae*. C. Detection limit on pooled and serially diluted *M. leprae*, *L. aethiopica*, and *M. tuberculosis* DNA samples. D. Clinical suspects; Lane 1: ladder, Lanes 5 and 16: *M. tuberculosis*; Lanes 4, 6, 7, and 8: *M. leprae*; Lanes 2, 3, 9, 10, 11, 12, 13, 14, 15, 17, and 18: L. aethiopica; Lane 19: negative control; Lane 20: positive control.

## Data Availability

Data are available upon request due to privacy/ethical restrictions.
