# Supplementary material for: Optimizing a Conventional Multiplex PCR for Simultaneous Detection of Granulomatous Skin Infection Agents: Leishmania aethiopica, Mycobacterium leprae, and Mycobacterium tuberculosis
Source: J Trop Med. 2026 Mar 11;2026:1456781. doi: 10.1155/jotm/1456781 (PMC12976814; doi:10.1155/jotm/1456781)
Supplement: Supplementary file 1 — Supporting Information 1 Supporting Table 1. List of primers utilized in this study (1A) and serial dilution of the analysis of the detection limits of the assay (1B). [file JOTM-2026-1456781-s001.docx]

| **List of DNA Primers Utilized in this Study** | | | | |  |
| --- | --- | --- | --- | --- | --- |
|  |  |  |  |  |  |
|  | **DNA** | **Primers Name** | **Sequence** | **Amplicon size** |  |
|  |  |  |  |  |  |
| 1 | *M.leprae* | RLEP-7: F | 5’-TGAGGCTTCGTGTGCTTTGC-3’ | 450 bp |  |
|  |  | RLEP-8: R | 5’-ATCTGCGCTAGAAGGTTGCC -3’ |  |  |
| 2 | *L. aethiopica* | LITSR: F | 5'-CTGGATCATTTTCCGATG-3' | 328 bp |  |
|  |  | L.5.8S: R | 5'-TGATACCACTTATCGCACTT-3' |  |  |
| 3 | *M. tuberculosis* | PT3: F | 5'-CACCACGTTAGGGATGCACTGC-3' | 223 bp |  |
|  |  | PT4: R | 5'-CTGATGGTCTCCGACACGTTCG-3' |  |  |
| 4 | Clinical sample | β-actin F | 5’ AGCGGGAAATCGTGCGTG 3’ | 304 bp |  |
|  |  | β-actin R | 5’ CAGGGTACATGGTGGTGC 3’ |  |  |

Supplementary table 1-A:

| **Serial Dilution of Characterized DNA Samples** | | | | | | | | | | |  |
| --- | --- | --- | --- | --- | --- | --- | --- | --- | --- | --- | --- |
|  |  |  |  |  |  |  |  |  |  |  |  |
| Tube | 1 | 2 | 3 | 4 | 5 | 6 | 7 | 8 | 9 | 10 |  |
|  |  |  |  |  |  |  |  |  |  |  |  |
| Titration | 1:2 | 1:4 | 1:8 | 1:16 | 1:32 | 1:64 | 1:128 | 1:256 | 1:512 | 1:1024 |  |
|  |  |  |  |  |  |  |  |  |  |  |  |
| Lane | 2 | 3 | 4 | 5 | 6 | 7 | 8 | 9 | 10 | 11 |  |
| Final DNA concentration | 5 ng | 2.5 ng | 1.25 ng | 0.63 ng | 0.31 ng | 0.16 ng | 0.08 ng | 0.04 ng | 0.02 ng | 0.01 ng |  |
|  |  |  |  |  |  |  |  |  |  |  |  |

Supplementary table 1-B
