# Supplementary material for: Optimizing a Conventional Multiplex PCR for Simultaneous Detection of Granulomatous Skin Infection Agents: Leishmania aethiopica, Mycobacterium leprae, and Mycobacterium tuberculosis
Source: J Trop Med. 2026 Mar 11;2026:1456781. doi: 10.1155/jotm/1456781 (PMC12976814; doi:10.1155/jotm/1456781)
Supplement: Supplementary file 2 — Supporting Information 2 Supporting Table 2. Diagnosis of the negative control group. [file JOTM-2026-1456781-s002.docx]

| **Diagnosis of the Control Samples** | | | |  |
| --- | --- | --- | --- | --- |
|  |  |  |  |  |
| **Category** | **Type of Skin Disorder** | **Frequency** | **Relative Frequency** |  |
| Malignant | Melanoma | 8.6% (n=6) | 18.6% (n=13) |  |
|  | Squamous Cell Carcinoma (SCC) | 2.9% (n=2) |  |  |
|  | Basal Cell Carcinoma (BCC) | 2.9% (n=2) |  |  |
|  | Sarcoma | 2.9% (n=2) |  |  |
|  | Parotid Cancer | 1.4% (n=1) |  |  |
| Benign | Chronic Wounds other than CL, TB, or leprosy | 14.3% (n=10) | 81.4% (n=57) |  |
|  | Post-burn Contracture | 10% (n=7) |  |  |
|  | Flap | 7.1% (n=5) |  |  |
|  | Bullet Injury | 4.3% (n=3) |  |  |
|  | Neurofibroma | 4.3% (n=3) |  |  |
|  | Ganglion Cyst | 2.9% (n=2) |  |  |
|  | Adenoma | 2.9% (n=2) |  |  |
|  | Deformity | 2.9% (n=2) |  |  |
|  | Others | 32.9% (n=23) |  |  |
