# Supplementary material for: Optimizing a Conventional Multiplex PCR for Simultaneous Detection of Granulomatous Skin Infection Agents: Leishmania aethiopica, Mycobacterium leprae, and Mycobacterium tuberculosis
Source: J Trop Med. 2026 Mar 11;2026:1456781. doi: 10.1155/jotm/1456781 (PMC12976814; doi:10.1155/jotm/1456781)
Supplement: Supplementary file 3 — Supporting Information 3 Supporting Table 3. Cross‐tabulation of the study and negative control groups. [file JOTM-2026-1456781-s003.docx]

|  |  | **Gold Standard** | | | **mPCR** | | |
| --- | --- | --- | --- | --- | --- | --- | --- |
|  |  |  |  |  |  |  |  |
| **mPCR** |  | **Positive** | **Negative** | **Total** | **Statistics** | **Value (%)** | **95% CI** |
|  | **Positive** | 47 | 0 | 47 | Sensitivity | 75.8% | 63.3% to 85.8 % |
|  | **Negative** | 15 | 70 | 85 | Specificity | 100% | 94.9% to 100 % |
|  | **Total** | 62 | 70 | 132 | PPV | 100% | 92.5% to 100 % |
|  |  |  |  |  | NPV | 82.4% | 75.0% to 87.9% |

Supplementary table 3. Cross-tabulation of study and negative control groups
