# Supplementary material for: Optimizing a Conventional Multiplex PCR for Simultaneous Detection of Granulomatous Skin Infection Agents: Leishmania aethiopica, Mycobacterium leprae, and Mycobacterium tuberculosis
Source: J Trop Med. 2026 Mar 11;2026:1456781. doi: 10.1155/jotm/1456781 (PMC12976814; doi:10.1155/jotm/1456781)
Supplement: Supplementary file 4 — Supporting Information 4 Supporting Table 4. Clinical data of study participants. [file JOTM-2026-1456781-s004.docx]

| **Cutaneous leishmaniasis participants (n= 45, 72.6%)** | | | | | | | |
| --- | --- | --- | --- | --- | --- | --- | --- |
| **Clinical Manifestation** | | | **Types of lesions** | | | | |
| LCL: n= 35 (77.8%) | MCL: n= 8 (17.8%) | DCL: n= 2 (4.4%). | Plaques: n=40 cases (88.9%) | crustations in n=27 (60%), | papules: n=25 (55.6%), | indurations n=12 (26.7%) | satellite papules  n= 6 (13.3%) |
| **Cutaneous leprosy participants (n= 9, 14.5%)** | | | | | | | |
| **Clinical Classification** | | **Types of Reaction** | | **Bacterial Index (BI)** | | | |
| PB: n=0 | MB: n=9 (100 %) | RR: n=5 (5.5 %) | ENL: n=1 (1.1%) | 1+: n= 1 (1.1%) | 2+: n=3 (1.5 %) | 3+: n=1 (1.1%) | 4+: n=4 (4.4%) |
| **Skin Tuberculosis participants (n= 4, 6.5%)** | | | | | | | |
| **Clinical Classification** | | **Types of lesions** | | | | | |
| Scrofuloderma (n=3, 75%). | TVC (n=1, 25%). | Induration and plaque (n=3, 75%), | | nodules and discharge (n=2, 50%). | | skin coloured (n=3, 75%) | erythematous (n=1, 25%), |
| **Potential coinfections (n= 4 ,6.5%)** | | | | | | | |
| CL and leprosy (n=2, 50%) | | | | skin tuberculosis and CL (n=2, 50%) | | | |

**Supplementary Table 4- Clinical Characteristics of Leprosy Participants (n=62)**
