# Supplementary material for: Optimizing a Conventional Multiplex PCR for Simultaneous Detection of Granulomatous Skin Infection Agents: Leishmania aethiopica, Mycobacterium leprae, and Mycobacterium tuberculosis
Source: J Trop Med. 2026 Mar 11;2026:1456781. doi: 10.1155/jotm/1456781 (PMC12976814; doi:10.1155/jotm/1456781)
Supplement: Supplementary file 5 — Supporting Information 5 Supporting Figure 1. Detection limits of the assay in potential double infections (1‐A), single infections (1‐B), and ITS‐1 product following digestion with the Hae‐III restriction enzyme (1‐C). [file JOTM-2026-1456781-s005.docx]

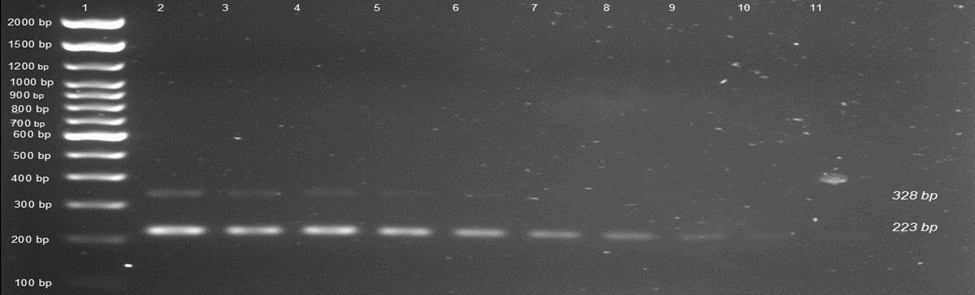


Supplemental figure 1-A: Agarose gel electrophoresis (1.5%) showing the assay's detection limit on *L. aethiopica* and *M. tuberculosis* DNA samples.


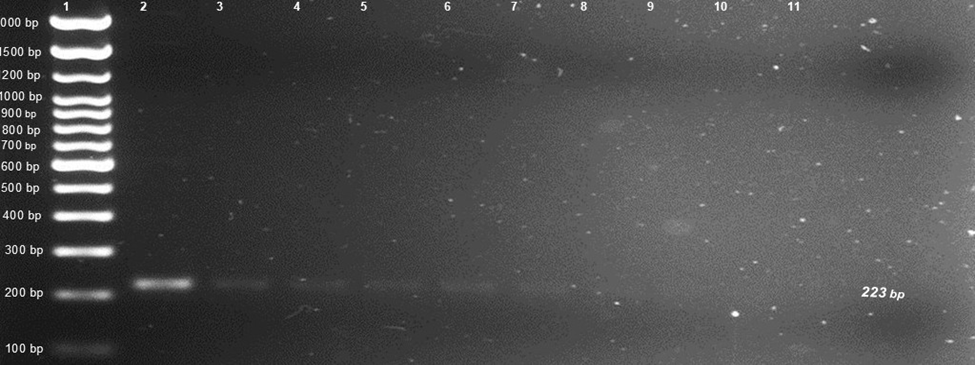


Supplementary figure 1-B: Agarose gel electrophoresis (1.5%) shows the detection limit of the assay for *M. tuberculosis.*


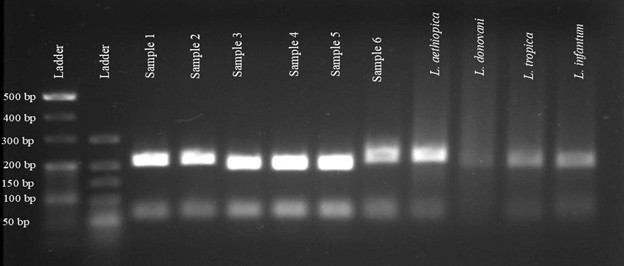


Supplementary figure 1-C: Agarose gel (2%) electrophoresis shows variations in band sizes 3, 4, and 5 of the leishmania species' ITS-1 product following digestion with the Hae-III restriction enzyme, and none of them resemble the four control reference strains.
