# Supplementary figures and images for: Optimizing a Conventional Multiplex PCR for Simultaneous Detection of Granulomatous Skin Infection Agents: Leishmania aethiopica, Mycobacterium leprae, and Mycobacterium tuberculosis
Source: J Trop Med. 2026 Mar 11;2026:1456781. doi: 10.1155/jotm/1456781 (PMC12976814; doi:10.1155/jotm/1456781)

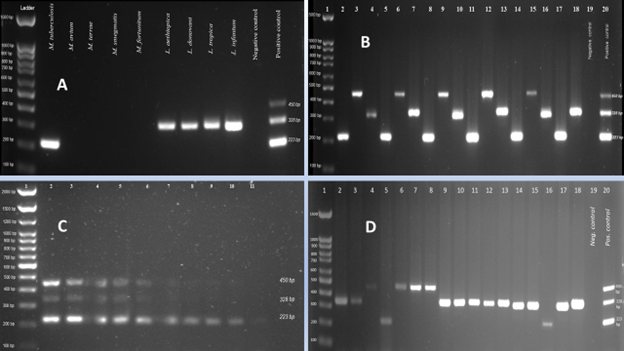

Supplement: Supplementary file 6 — Supporting Information 6 Supporting Figure 2. Agarose gel electrophoresis (1.5%); A. Specificity of the assay on Mycobacterial and Leishmania reference strains. B. Characterized DNA samples of M. tuberculosis, L. aethiopica, and M. leprae. C. Detection limit on pooled and serially diluted M. leprae, L. aethiopica, and M. tuberculosis DNA samples. D. Clinical suspects; Lane 1: ladder, Lanes 5 and 16: M. tuberculosis; Lanes 4, 6, 7, and 8: M. leprae; Lanes 2, 3, 9, 10, 11, 12, 13, 14, 15, 17, and 18: L. aethiopica; Lane 19: negative control; Lane 20: positive control. [file JOTM-2026-1456781-s006.tif]
